# Retraction: Arid3b Is Critical for B Lymphocyte Development

**DOI:** 10.1371/journal.pone.0309551

**Published:** 2024-08-22

**Authors:** The PLOS ONE Editors

After this article [1] was published, concerns were raised about Fig 6B.

Specifically:

- Lanes 1–8 in Fig 6B appear similar to the left 8 lanes of Fig 5.3A of [2] and Fig 4A of [3], but are labeled differently.
- Lanes 9–14 in Fig 6B appear similar to the right 6 lanes of Fig 5.3B of [2] and Fig 4B of [3], but are labeled differently.

Of note, the author of [2] is not in the *PLOS ONE* article’s author list or otherwise acknowledged in [1].

The University of Texas at Austin investigated this matter and concluded that Fig 6B of [1] reports falsified data. The University of Texas at Austin advised that Fig 6 was the only figure supplied by University of Texas collaborators; the article’s other results were not reviewed in their investigation.

In reviewing this matter, PLOS noted that the primary data were not provided with the published article [1] contrary to the Data Availability statement. The authors stated that not all of the underlying data for this article remain available. Therefore, this article does not comply with the PLOS Data Availability policy.

The *PLOS ONE* Editors retract this article due to the falsification of results reported in Fig 6B.

WMH, RR (formerly RAS), KDCD, and RD agreed with the retraction, apologized for the issues with the published article, and stand by the article’s findings. HT did not agree with the retraction and apologized for the issues with the published article. JK, NK, CW and CD either could not be reached or did not respond directly.

Fig 6B reports material from [2], published in 2004 by the University of Texas at Austin, which is not offered under a CC BY license and is therefore excluded from this article’s [1] license. At the time of retraction, the article [1] was republished to note this exclusion in the Fig 6 legend and the article’s copyright statement.
