# Correction: Retraction: Arid3b Is Critical for B Lymphocyte Development

**DOI:** 10.1371/journal.pone.0310392

**Published:** 2024-09-09

**Authors:** The PLOS ONE Editors

The *PLOS ONE* Editors issue this notice to correct an error in the retraction notice [1] for this article [2]. Contrary to the statement in the retraction notice, the article [2] was not republished at the time of retraction but instead was republished on August 28, 2024. The article’s [2] Copyright statement and Fig 6 legend have now been updated to note the exclusion of Fig 6B from this article’s license.

The publisher apologizes for the error.
